# Wire-Based Additive Manufacturing of Ti-6Al-4V Using Electron Beam Technique

**DOI:** 10.3390/ma13153310

**Published:** 2020-07-24

**Authors:** Florian Pixner, Fernando Warchomicka, Patrick Peter, Axel Steuwer, Magnus Hörnqvist Colliander, Robert Pederson, Norbert Enzinger

**Affiliations:** 1 Institute of Materials Science, Joining and Forming, Graz University of Technology, Kopernikusgasse 24, 8010 Graz, Austria; florian.pixner@tugraz.at (F.P.); fernando.warchomicka@tugraz.at (F.W.); patrick.peter@liebherr.com (P.P.); 2 Liebherr-Werk Telfs GmbH, 6410 Telfs, Austria; 3 Nelson Mandela University, Port Elizabeth 6031, South Africa; axel.steuwer@um.edu.mt; 4 Research Support Services, University of Malta, 2080 Msida, Malta; 5 Department of Applied Physics, Chalmers University of Technology, 41296 Göteborg, Sweden; magnus.colliander@chalmers.se; 6 Department of Engineering Science, University West, 46132 Trollhättan, Sweden; robert.pederson@hv.se

**Keywords:** additive manufacturing, titanium alloys, electron beam, wire feed process, residual stresses, mechanical properties

## Abstract

Electron beam freeform fabrication is a wire feed direct energy deposition additive manufacturing process, where the vacuum condition ensures excellent shielding against the atmosphere and enables processing of highly reactive materials. In this work, this technique is applied for the α + β-titanium alloy Ti-6Al-4V to determine suitable process parameter for robust building. The correlation between dimensions and the dilution of single beads based on selected process parameters, leads to an overlapping distance in the range of 70–75% of the bead width, resulting in a multi-bead layer with a uniform height and with a linear build-up rate. Moreover, the stacking of layers with different numbers of tracks using an alternating symmetric welding sequence allows the manufacturing of simple structures like walls and blocks. Microscopy investigations reveal that the primary structure consists of epitaxial grown columnar prior β-grains, with some randomly scattered macro and micropores. The developed microstructure consists of a mixture of martensitic and finer α-lamellar structure with a moderate and uniform hardness of 334 HV, an ultimate tensile strength of 953 MPa and rather low fracture elongation of 4.5%. A subsequent stress relief heat treatment leads to a uniform hardness distribution and an extended fracture elongation of 9.5%, with a decrease of the ultimate strength to 881 MPa due to the fine α-lamellar structure produced during the heat treatment. Residual stresses measured by energy dispersive X-ray diffraction shows after deposition 200–450 MPa in tension in the longitudinal direction, while the stresses reach almost zero when the stress relief treatment is carried out.

## 1. Introduction

Additive manufacturing (AM) comprises different processes which deal with different materials using different heat sources to build-up structural parts. The ISO 17296-2:2015 standard provides an overview of existing AM process categories [1]. Processes can be classified according to their general principles as follows: vat photopolymerization, material jetting, binder jetting, powder bed fusion (PBF), material extrusion, directed energy deposition (DED), and sheet lamination.

For reactive materials like Ti-based alloys, the number of feasible processes is limited and the demands on the shielding environment are remarkably high to avoid atmospheric contaminations. Nowadays Ti-6Al-4V, recognized as the most popular α + β titanium alloy, is processed by various AM processes like powder-based processes, selective laser melting (SLM), electron beam melting (EBM), or wire-based DED techniques. The respective processes show certain characteristics and result in intrinsic properties of the manufactured parts. Powder-based processes are very common for AM and are leading technologies performing complex geometries and surface finishing. In AM, there is a correlation and trade-off between maximum resolution and achievable deposition rates. Due to the usage of powder in the micrometer range, the achievable deposition rates are limited, and the part size is restricted to the dimensions of working chambers. Wire-based DED processes widen the field of application and have received considerable attention due to printing of more volumetric structures with simultaneous high deposition rates. Electron beam freeform fabrication (EBF³, EBF3, or EBFFF) is based on a wire feed DED-process using the electron beam as heat source and the suitability for processing titanium has already been demonstrated [2,3,4,5]. Since electron beam technique is based on a vacuum atmosphere (<5 × 10^−3^ mbar), it is suitable to process reactive materials like titanium. The process can be described as a near-net-shape manufacturing process with a high deposition rate (up to 2500 cm^3^/h) which usually requires an additional final processing step, e.g., subtractive manufacturing [6]. The characteristic high-power density of the electron beam welding (EBW) process of up to 10^7^ W/cm^2^ is not required in case of AM, because there is no need of a deep penetration since dilution should be minimized [7]. This type of process is currently used for commercial purposes to produce robust structures of different titanium, tungsten, and inconel alloys, among others [8].

In the case of wire-based electron beam additive manufacturing (EBAM), there are numerous technological input parameters—e.g., (1) acceleration voltage, (2) beam current, (3) welding speed, (4) wire feed rate, (5) beam figure, and (6) focus position—which can be varied in a wide range and a proper selection of them can be challenging [9]. Another factor in process design is the way of the wire-feeding. Usually, wire arc additive manufacturing (WAAM) processes use an electric arc as energy source without electromagnetic force between the molten droplet and the substrate plate. In the case of wire-based electron beam additive manufacturing, the filler wire input is independent from the energy input and, for this reason, the parameters for material input and energy can only be varied independently to a limited extent. A proper height alignment of the wire tip with the electron heat source is necessary for the material transfer and a stable melt pool [10]. Since the melt pool and melt pool temperature can be controlled by process parameters [11,12,13], and weld beads are formed during solidification, knowledge about the correlation between process parameters and weld bead geometry is essential for process control and to understand the fundamentals of EBAM. Starting from weld bead geometry and knowledge of single bead profiles opens the capability for finding an optimum overlapping distance and is an important input for the subsequent welding sequence and to build up more complex geometries [14].

The mechanical properties during static and dynamic loading are influenced mainly by (1) the segregation of elements, (2) volume defects and (3) the microstructure [15,16]. During additive manufacturing, defects are mainly related to porosity affected by the process parameters and the type of adding material [17,18,19], cracks formation due to residual stresses generated during the process and delamination due to incomplete melting [18]. Insufficient bonding—e.g., lack of fusion as the major defect—has a negative effect on the static and dynamic mechanical properties of AM parts [20,21]. The variety of microstructure has a significant role in the mechanical properties of components made by titanium alloys [22,23]. In the case of additive manufacturing processing, the microstructural features (prior β grains, martensite, and α phase morphology, among others) depend on the experienced thermal cycle provoked by the type of process. Zhang et al. [24] summarized the progress in powder-based EBM in the field of α + β and β titanium alloys for solid and porous components, describing not only the microstructure and the mechanical properties, but also the unfavorable issues in the process and complex thermal conditions. The accumulation of layers and multiple thermal cycles in prevailing vacuum atmosphere can lead to heat accumulation and low cooling rates in EBM [24]. SLM produce mostly martensitic structure due to the cooling rate higher than 1000 K/min [19,22,25,26] reached in the layering. This microstructure can be decomposed in two phases (α + β) by post heat treatment [19] or by thermal cycling during layer-by-layer building (i.e., EBM [24,27,28,29]), obtaining fine lamellar, colony, or Widmanstätten α structure and small amount of β phase [15]. For processes with high deposition rate and energy, the cooling rates are lower and it can form directly α + β in basket weave Widmanstätten or lamellae α morphology depending on the previous build part (e.g., [19,30,31,32,33,34,35]).

Recent literature [3,4] is limited for electron beam additive manufacturing, where the emphasis is mainly on material properties and microstructural details, and not on the process and parameter selection itself. The present study aims to establish suitable parameters for the EBAM process in α + β titanium alloy Ti-6Al-4V and determine the influence of these parameters on the dimensions of the single and multi-tracks welds for building walls with an optimal sequence of building. Furthermore, the microstructure and residual stresses produced during the manufacturing are analyzed, and the mechanical properties are estimated for the as-deposited and stress relief heat treated conditions.

## 2. Materials and Methods

### 2.1. Materials

Titanium alloy wire AWS A5.16 ER Ti5 (EN ISO 24034) was used with a commercially available diameter of 1.2 mm. The deposition was carried out with two different substrates: Commercial pure Titanium Grade 2 for the parameter studies and the single bead experiments; and Titanium Grade 5 (Ti-6Al-4V) for the subsequent multilayer-experiments, and mechanical as well as metallographic characterization.

The chemical compositions of the applied titanium alloy wire and the AM structures made out of it were determined as follows: the proportion of the elements aluminum (Al), vanadium (V), and iron (Fe) are determined by atomic absorption spectroscopy. The interstitial elements carbon (C) via solid-state infrared absorption detection method with LECO CS230 (LECO Corporation, St. Joseph, MI, USA); nitrogen (N) via thermal conductivity detection method with LECO ON 736 (LECO Corporation, St. Joseph, MI, USA); and oxygen (O) via non-dispersive infrared absorption detection method with LECO TCH600 (LECO Corporation, St. Joseph, MI, USA). Table 1 shows the chemical composition of the applied wire in this work.

For the titanium alloy wire, the proportion of all elements with the exception of vanadium is according to the specification. Only the proportion of vanadium is slightly below the permissible lower limit of the specification.

### 2.2. Experimental Setup

All welding experiments were performed using the electron beam welding machine pro-beam EBG 45-150 k14 (Probeam GmbH & Co. KGaA, Gilching, Germany). The pressure at the working chamber was below 5 × 10^−3^ mbar. Inside the chamber, with a nominal volume of about 1.4 m^3^, a three-axis working table was used to handle the substrate plate. The welding filler wire was fed by pressure rollers and guided through a polytetrafluoroethylene (PTFE) hose and ends in an in three-axis movable water-cooled wire nozzle. The angle between the electron beam and the fed wire was 55°, and the distance of the nozzle to the substrate surface was 8 mm. The selected alignment was a trade-off between a proper wire guidance and a certain distance to avoid overheating of the nozzle during the process. Furthermore, a height distance of 1 mm between the wire tip and the substrate was set to ensure a continuous liquid metal bridge and thus a stable metal transfer during the process.

The main process relevant input and beam oscillation parameters are listed in Table 2. The acceleration voltage *U_acc_* was kept constant and the power input was adjusted by varying the beam current *I_beam_*. In order to adapt the power density, a customized beam figure consisting of several concentric circles with defined radii and elements was applied. A maximum outer diameter of 4 mm was chosen. In addition, the welding speed *v_weld_* (9.0–11.0 mm/s) as well as the wire feed rate *v_wire_* (2.7–3.9 m/min) were altered for the design of experiment (DOE) approach.

Two full factorial DOE’s (Table 3) were carried out in order to evaluate the parameter influence on the weld bead width and height of the deposited single beads. The analysis of the dilution was done with a fractional factorial design in the moderate power range (Table 3). The obtained data was statistically evaluated, analyzed, and visualized by the means of the software MiniTab 19 (Version 19.1, Minitab, LLC, State College, PA, USA).

### 2.3. Heat Treatment

The conducted heat treatment was selected according to literature. The post weld heat treatment (PWHT) has the purpose of soft-annealing and stress relieving. It was done in the preheated furnace at atmosphere for 2 h at 710 °C [15,36,37] and cooled in still air. The formation of alpha case during the heat treatment was irrelevant and any indication of oxidation at the surface was removed for subsequent mechanical characterization.

### 2.4. Metallographic Characterization

Macroscopic analysis was conducted by the means of a stereo microscopy (Zeiss Discovery V20, Zeiss, Oberkochen, Germany) to observe the strategy of building and defects related to the process (lack of fusion, pores, etc.). Microstructure and the effect of the process parameter on the dilution of single beads and overlapping distance for multi-track building was analyzed by a light optical microscope (Zeiss Axio Observer Inverted, Zeiss, Oberkochen, Germany). The geometry of the weld beads was measured by the related image processing program AxioVision 4.8.2 (Zeiss, Oberkochen, Germany).

Field emission scanning electron microscopy (SEM, TESCAN Mira 3, Tescan, Brno, Czech Republic) was used for characterization of the final microstructure after processing and heat treatment.

The examined embedded cross-sections were immersed in a Kroll’s reagent etchant with a solution of 2 mL hydrofluoric acid (40%), 4 mL nitric acid (65%), and 94 mL distilled water. The etching duration was in the range of 3–40 s, observing a faster effect of the etchant on heat treated samples.

### 2.5. Mechanical Characterization

The mechanical properties of the AM structures were evaluated by tensile tests, Charpy V-notch impact tests, and hardness measurements at room temperature (20 °C).

The tensile testing specimens (DIN 50125-C, 6 mm diameter × 30 mm gauge length) were machined out of the block structures and are orientated along the welding direction. Therefore, the tensile properties were determined only in the longitudinal direction for the as-deposited and PWHT-condition due to the limited volume of processed material. The tensile tests were performed according to DIN EN ISO 6892-1 [38], with a constant testing speed of 1 mm/min on tensile testing device Zwick RMC 100 (ZwickRoell, Ulm, Germany).

**Figure 1:**
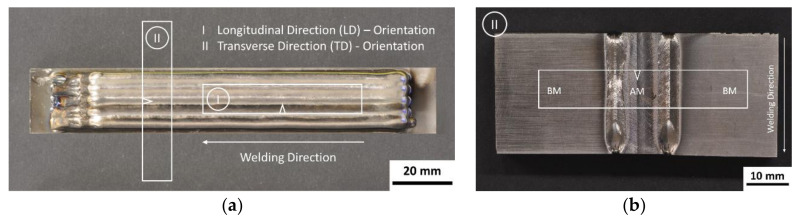
Charpy V-notch specimens: (**a**) orientation of the specimens in the AM blocks; (**b**) electron beam welded transverse Charpy V-notch specimen.

Charpy V-notch impact tests were carried out according to DIN EN ISO 148-1:2017 [39]. The impact energy was measured for a longitudinal and transversal oriented samples in relation to the welding direction for the as-deposited and PWHT-condition (Figure 1a). The V-notch is located perpendicular to the sample orientation and the recorded absorbed energy represents the actual impact energy rectangular to the actual orientation. Therefore, the longitudinal orientated sample represents the properties across the welding direction, whereas the transverse sample represents the properties along the welding direction.

To determine the impact toughness along the welding direction according to the DIN EN ISO 148-1:2017 [39] standard and required dimensions, a novel approach was chosen. A trimmed transverse AM inlay was electron beam welded to parent material cantilevers (Figure 1b). The characteristic high energy density provided by EBW process results in a narrow weld zone. It might be assumed that the actual fracture area and the related recorded impact energy are not influenced by the previous EB welds.

Vickers hardness measurements according to DIN EN ISO 6507-1:2016 [40] were performed by using a load of 0.5 kgf (4.903 N) and a dwell time of 15 s (HV 0.5). The measurements were carried out on EMCO M1C hardness testing device (EMCO-Test, Kuchl, Austria). The hardness distribution of the AM cross-sections was obtained by hardness mapping visualized by the software Origin (Version 8.6, OriginLab Corporation, Northampton, MA, USA) and line scans for the as-deposited and PWHT conditions.

### 2.6. Residual Stress Measurements in Single Wall

The residual stress measurements in one single-track multilayer EBAM wall were carried out by energy dispersive X-ray diffraction (EDXRD) on the high-energy beam line ID15A at the European Synchrotron Research Facility (ESRF) in Grenoble, France. The characterization used a high-flux white beam with an energy range of 50–250 keV (wavelength range of 0.2480–0.0496 Å). Measurements were done in transmission mode with a slit size of 100 × 100 µm^2^, giving a diamond shaped gauge volume with a length of approximately 2 mm. Diffraction spectra were collected by two energy-discriminating detectors placed at diffraction angles of 2θ = 5° in the horizontal and vertical direction, allowing determination of strains in two directions simultaneously. Three parallel lines at the middle of the wall length were measured, spaced 2 mm apart to obtain a good reproducibility. A total of 10 points per line were acquired from the bottom (interface between the wall and the substrate) to the upper part with 1.6 mm space between the points. Each point was measured for 60 s, and during the measurement the sample was moved back and forth ±1 mm to increase statistics. Pawley refinement using GSAS (General Structure Analysis System) [41] was used to extract the lattice parameters (a and c) of the α phase in both directions. The low volume fraction and strong texture prevented the lattice parameter of the β phase to be determined reliably. The same AM build was measured before and after PWHT for 2 h at 710 °C.

## 3. Results

### 3.1. Single Layer Experiments

#### 3.1.1. Dimension of Single Beads

**Figure 2:**
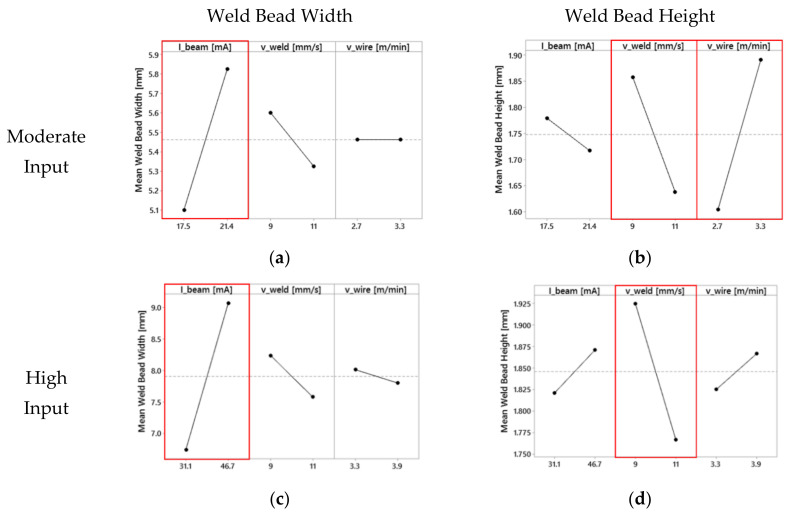
DOE analysis: Influence of process parameters on the weld bead geometry at (**a**,**b**) moderate and (**c**,**d**) high input, and related significant process parameters are framed red.

The relation of the process parameters on the geometric evolution of the deposited weld beads by two full factorials DOEs is illustrated in Figure 2. The main effect plot visualizes the consequence of the predefined factors *I_beam_*, *v_weld_*, and *v_wire_* on the target values: (1) weld bead width b and (2) weld bead height h. Considering the analyses’ main effects on magnitude and slope, the single strength of the effects for the selected input factors of the moderate and the high input DOE show a good agreement with the literature [9]. The steeper the slope of the line, the greater is the impact of the factor on the geometry of the single beads. For both type of input (factorials), the increment of the *I_beam_* increases the width of the bead with a slight change in the height, while the increase of the *v_weld_* produced lower and narrower weld beads due to reduced energy input and material input per unit length. The wire feed rate *v_wire_* has a strong influence on the weld bead height during processing at moderate input. The bead height increases by increasing the *v_wire_* with a negligible change in the width (nearly horizontal line in Figure 2a). For high input, there are not significant changes of the bead geometry by varying the *v_wire_*.

#### 3.1.2. Dilution of Single Bead

**Figure 3:**
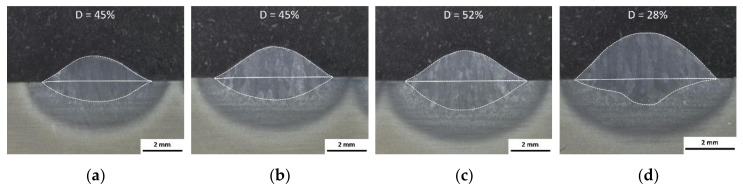
Dilution and geometric evolution of single beads deposited with different parameter configurations given in Table 4. Dilution: (**a**,**b**) 45%, (**c**) 52% and (**d**) 28%.

The weld bead dilution D is defined by the ratio between the molten base material (BM) and the whole weld bead cross section [42]. A minimum dilution leads to produce an efficient and fast build-up, and it would be reached by low energy per unit length and high deposition rate. In this work, the parameters analyzed by DOE for moderate heat input (Figure 2a,b; Table 3) are used for the dilution estimation. The analysis of the dilution was done with a fractional factorial design, and Table 4 summarizes the parameter used for the single bead. The ratio of wire feed rate to welding speed represents the material input per length (*v_wire_/v_weld_*) and amount of weld bead reinforcement. Figure 3 shows the cross section and dilution obtained for each parameter.

Figure 3a,b shows the same dilution of 45% for different applied process parameters. Therefore, different process parameter configurations may be possible to achieve the same dilution, which should be minimized.

From Figure 3b to Figure 3c, not only the welding speed is reduced from 11 to 9 mm/s, but also the wire feed rate is reduced from 3.3 to 2.7 m/min. It results in a constant ratio of wire feed rate to welding speed, which corresponds to a constant material input per length value of 5.0. Since the welding speed (energy input per length) has a weak influence on the weld bead width (Figure 2a,c), more base material is fused by decreasing the welding speed from 11 to 9 mm/s (Figure 3b,c). By increasing the area of fused base material and keeping the material input per length constant, the dilution consequently increases from 45% to 52%.

A reduction of the beam current from 21.4 mA (Figure 3c) to 17.5 mA (Figure 3d) results in less area of the molten base material. By simultaneously increasing the wire feed rate, hence material input per length, the dilution can be optimized to a minimum value. Observations show a minimum dilution of 28%, and this particular configuration of the parameters is used for subsequent experiments.

#### 3.1.3. Overlapping Distance for Multi Track

**Figure 4:**
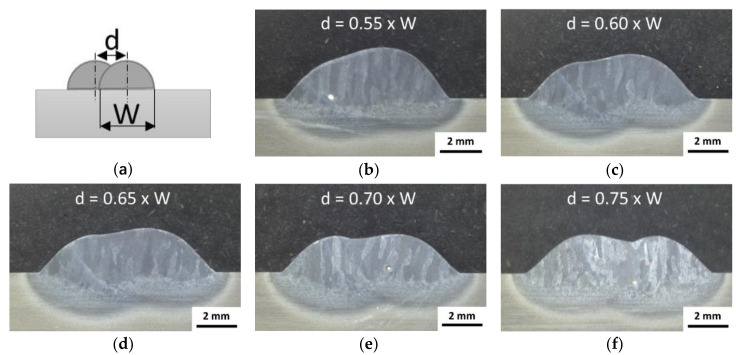
Multi-track overlapping distances (ratio d/W): (**a**) scheme of the overlapping with the main parameters d and W, (**b**) 0.55, (**c**) 0.60, (**d**) 0.65, (**e**) 0.70, and (**f**) 0.75.

The production of structures by EBAM requires the optimization of the overlap of the beads for a uniform and flat surface for successive layers during the build-up process. The overlapping distance given by the axial offset (d) is linked to the width (W) of the single bead, as shown in Figure 4a. Figure 4b–f show for different ratios (d/W) the form and symmetry of the deposited overlapped beads. If the overlapping distance is too small (e.g., ratio 0.55) an asymmetric layer is observed. The increment of the distance helps for the symmetry in the overlapping, with even surface up to ratio of 0.75, where wavelike surface with valleys in between individual beads is observed (Figure 4f). A compromise overlapping distance was identified in the range of 70–75% of the bead-width, and is in good agreement with Suryakumar et al. (66.6%) [43] or Ding et al. (73.8%) [44] reported for gas metal arc welding (GMAW) process. The manufacturing of the AM structures in the following sections is done with an axial offset of 4 mm between the deposited tracks, which means an overlapping distance of about 72%.

### 3.2. Building AM Block

**Figure 5:**
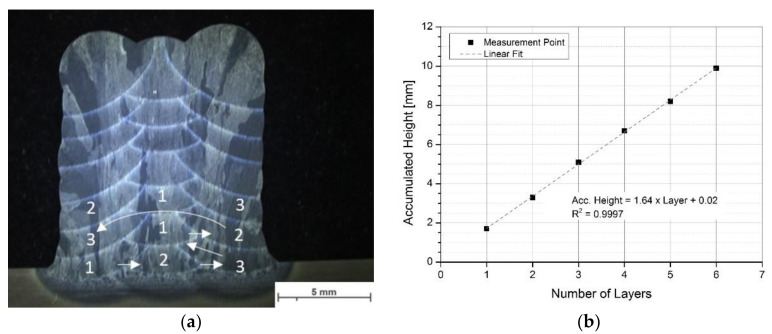
(**a**) Alternating symmetric welding sequence to build-up rectangular AM structures and (**b**) measured accumulated height over number of layers and ascertain growth rate by the means of linear fit.

AM blocks were built by multi-track and multi-layer production, using optimized parameters in Section 3.1. The power input was steadily decreased (approx. 0.5 mA after every second layer) by increasing the number of layers to compensate the preheating effect of prior layers and the consequently reduced heat flux with increasing height. Figure 5a shows the transverse cross-section of the built AM block after seven layers with a linear growth rate of the height by adding continuously layers (Figure 5b). The derived linear fit indicates an average growth rate of about 1.64 mm for each layer that helps to set up an automated process without needs of venting the chamber.

**Figure 6:**
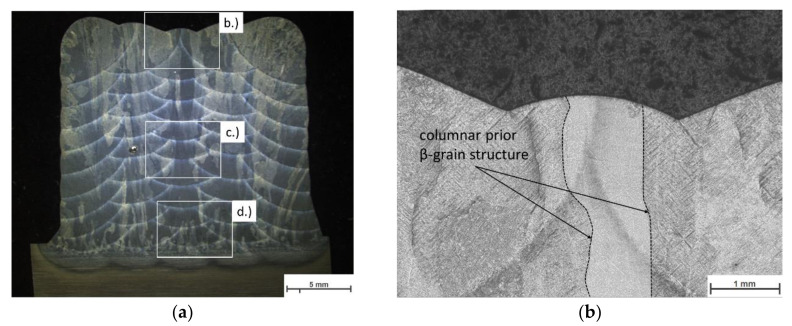

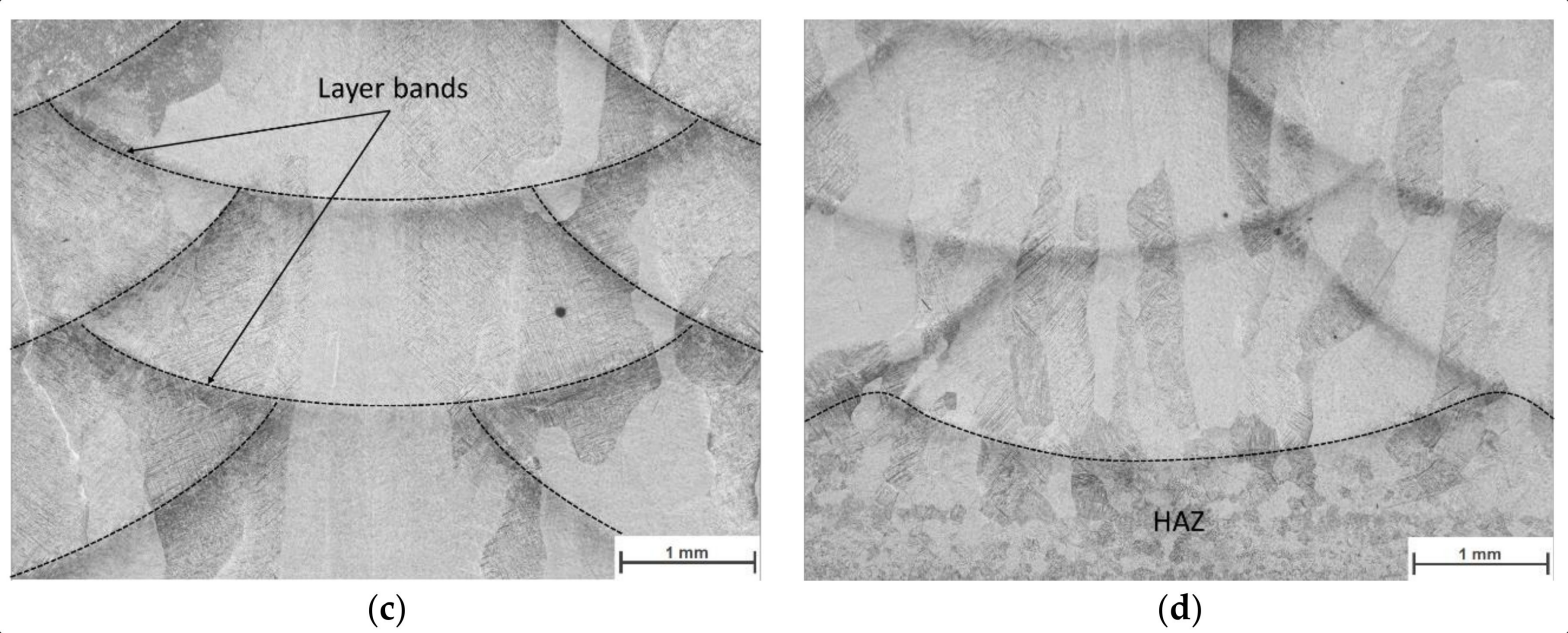
(**a**) Macrostructure with layer bands and columnar structure, (**b**) columnar prior β-grain structure, (**c**) presence of several layer bands, and (**d**) transition AM bulk material to heat-affected zone (HAZ).

According to literature [14,32,45,46,47,48,49,50,51], different welding sequences are reported and well established for AM processes. An alternating symmetrical welding sequence permits a robust process design for rectangular structures with parallel walls (Figure 5a and Figure 6a). For that, the first bead must be welded in the center of the previous layer and the subsequent beads follow from inside out, alternating on both sides (see marks in Figure 5a).

### 3.3. Chemical Composition AM Block

The chemical compositions of the studied AM blocks are given in Table 5. The chemical composition of the AM material shows only evaporation losses of aluminium in the range of approx. 1 wt % (approx. 14%). The measured oxygen concentration of 0.11 wt % is within the specification and indicates no further oxygen pick-up by the ambient vacuum atmosphere or contamination.

Since aluminum is the element in Ti-6Al-4V with the highest saturated vapor pressure, it can be expected that it has a significant tendency to evaporation and vaporization loss during processing. The evaporation of aluminum by processing Ti-6Al-4V with different AM processes has already been reported in the literature (i.e., EBF^3^ [4,52], E-PBF [53,54], or L-PBF [55]). Unlike L-PBF, the excessive aluminum loss was reported for vacuum based processes E-PBF (up to 30%) and EBF^3^ (up to 39%) by Juechter et al. [54] and Xu et al., respectively [4]. By minimizing the dilution, the excessive re-melting of previous layers and additional evaporation of aluminum can be avoided. The evaporation loss of aluminum in the present study is significantly lower compared to the investigations of Xu et al. [4].

### 3.4. Metallography

Metallographic investigations were performed on cross-sections of AM blocks built by 10 layers × 5 beads, with an alternating symmetric welding sequence (Figure 6a).

The AM cross-section shows epitaxial grown columnar prior β-grains, reaching over several layers and parallel to the build direction. The form and size of the columnar prior β-grains is provoked by the large temperature gradient during the solidification in direction of the heat flow [56,57]. A very low amount of macro and micro pores with a random distribution is observed along the section. Layer bands (Figure 6a,c) represent a minimal change of the microstructure (HAZ) due to the thermal cycles and intrinsic heat treatment of the neighbored weld bead deposition during the building process, as observed as a dark zone by etching effect in wire based investigations [33,56,58,59]. Recent work has demonstrated a minimal segregation near to these layers, mainly related to β-stabilizer elements [59]. The transition between the AM-Structure and the substrate consists in coarser equiaxed prior β-grains (Figure 6d).

**Figure 7:**
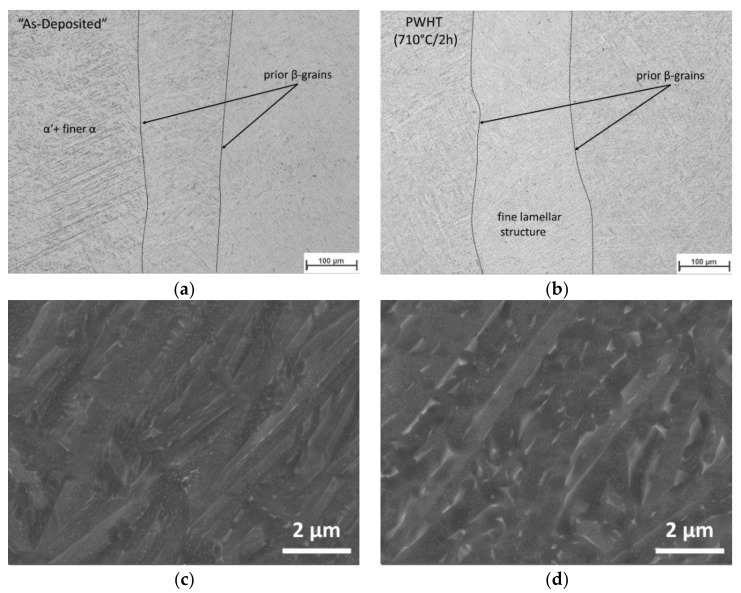
Microstructure of the AM block center: (**a**) as-deposited and (**b**) after PWHT condition; Detail of the microstructure by SEM investigations in (**c**) as-deposited and (**d**) after PWHT conditions.

The fast cooling rates reached during the process (see Supplementary Data, Figures S1 and S2) promote the formation of a mixture microstructure of finer α and martensite (α’) along the whole AM structure, as shown in Figure 7a,c. The morphology of the microstructure is almost homogenous in the whole building block. After the PWHT, the microstructure decomposes into in α + β structure, with a fine α-lamellar structure (Figure 7b). Precipitation of fine β within the fine α lamella detected in the Figure 7d can be probably due to an heterogeneous enrichment of β-stabilizer elements [60].

### 3.5. Residual Stresses by High Energy Dispersive X-ray Diffraction

**Figure 8:**
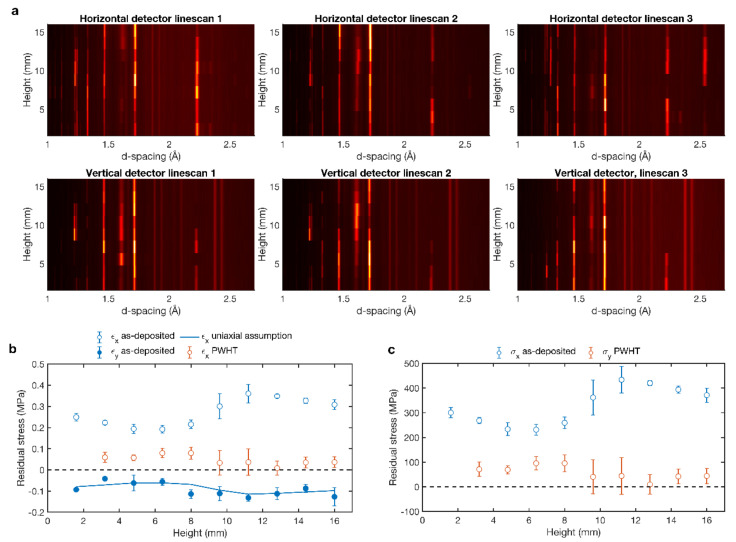
(**a**) 2D plot diffraction spectra of the as-deposited condition, for the horizontal and vertical detectors as a function of height in the wall. (**b**) Residual strains and (**c**) residual stresses for as-deposited and PWHT conditions as a function of height in the single wall.

Diffraction spectra in both directions for the three different linescans is illustrated in Figure 8a as 2D-plot (position vs. d-spacing) for as-deposited condition. The combination of large grains, strong texture and parallel X-ray radiation results on variations across the height, where certain peaks appear and disappear in in the spectra. This resulted in d-spacing errors in the range of (50–100) × 10^−6^ and (100–150) × 10^−6^ for a and c lattice parameters, respectively, from refinements. In the following, results from all three linescans were averaged.

The information obtained by both detectors helps to determine the spatially resolved residual strains and stresses. Strains are calculated as function of the lattice parameter a and c [61]

ε = (2 × ε_a_ + ε_c_)/3, (1)

where,

ε_a_ = a/a_0_ − 1, (2)

ε_c_ = c/c_0_ − 1, (3)

Reference lattice parameters are calculated from the average of the values measured in the vertical direction in the PWHT condition due to the constant value observed in all the positions. a_0_ is 2.92612 Å and c_0_ is 4.67067.

Figure 8b shows the calculated residual strains in the *x*- and *y*-direction for the as-deposited sample. Generally, the all three principal strain need to be considered in order to calculate the corresponding stresses, which requires measurements of strains also in the z-direction. However, as seen in Figure 8b there is an excellent agreement between the measured ε_y_ and the values calculated as—ν. ε_x_ based on the assumption of a uniaxial stress in the x-direction (where ν = 0.317 is Poisson’s ratio [21]). Consequently, only strain and stresses in the *x*-direction are considered hereafter. Also included in Figure 8b is ε_x_ for the PWHT condition, which shows significantly slower strain levels.

Under the assumption of a uniaxial stress state, the stress–strain relationships simplify to

σ_x_ = E × ε_x_, (4)

and σ_y_ = σ_z_ = 0. The Young’s modulus, E, for this alloy is 120.4 GPa [21]. Figure 8c shows the resulting residual stresses as a function of height in the wall. The error bars represent the scatter obtained from averaging the results from the individual linescans at each position. Residual stresses lie in the range of 200–450 MPa in tension for the *x*-direction, which is a result of the thermal contraction of the deposited material. The trends agree well with previous measurements and process simulations of AM Ti-6Al-4V walls [62]. After annealing, the stresses are lower than 100 MPa and closer to zero in the upper part of the single wall.

### 3.6. Mechanical Properties

#### 3.6.1. Tensile Test

**Figure 9:**
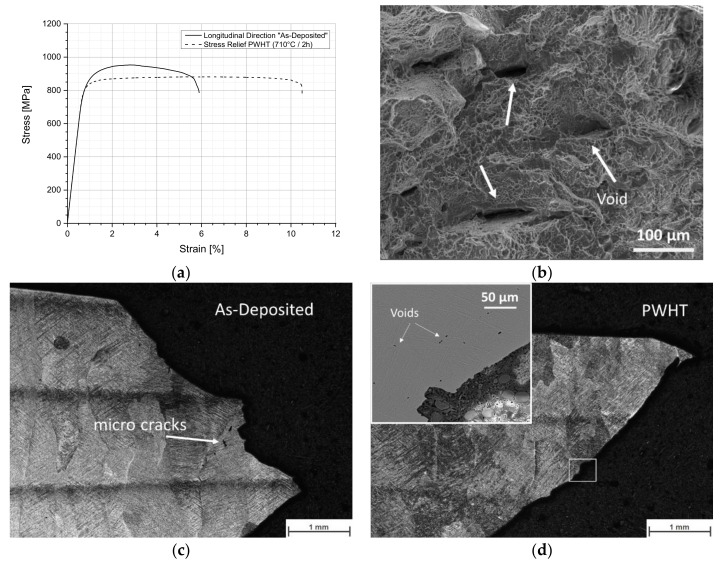
(**a**) Engineering stress–strain curves for longitudinal orientated tensile specimens. (**b**) Detail of the fracture surface for the PWHT condition. (**c**,**d**) Macrograph of the cross section of the fracture area of as-deposited and PWHT conditions, respectively.

The tensile stress over strain curves for the longitudinal orientated specimens in the as-deposited and PWHT condition are presented in Figure 9a. The as-deposited condition has a yield tensile strength (YS) of 846 MPa, ultimate tensile strength (UTS) of 953 MPa, and a fracture elongation (El) of 4.5%. When the heat treatment (PWHT) is carried out, a slight reduction of the YS and UTS values is observed but the El increases by more than 100% up to 9.5%.

According to the specifications for wrought material Ti-6Al-4V (YS > 795 MPa, UTS > 860 MPa, and El > 10%) [22,63], the tensile strength of the AM block structure in as-deposited condition is on the lower limit of the specification limit and the low fracture elongation might be related to the type of cast material. The fractured surface (Figure 9b) shows a ductile morphology, denoted by transgranular dimples and microcracks formed by coalescence of voids. This observation is similar to materials made by WAAM process [34]. The nucleation of voids is observed at the α/α’ and β phases interface, and they start to grow and coalesce along with the interface. Example of voids and microcracks are illustrated in Figure 9c and d for as deposited and PWHT conditions, respectively.

#### 3.6.2. Charpy V-Notch Impact Tests

**Figure 10:**
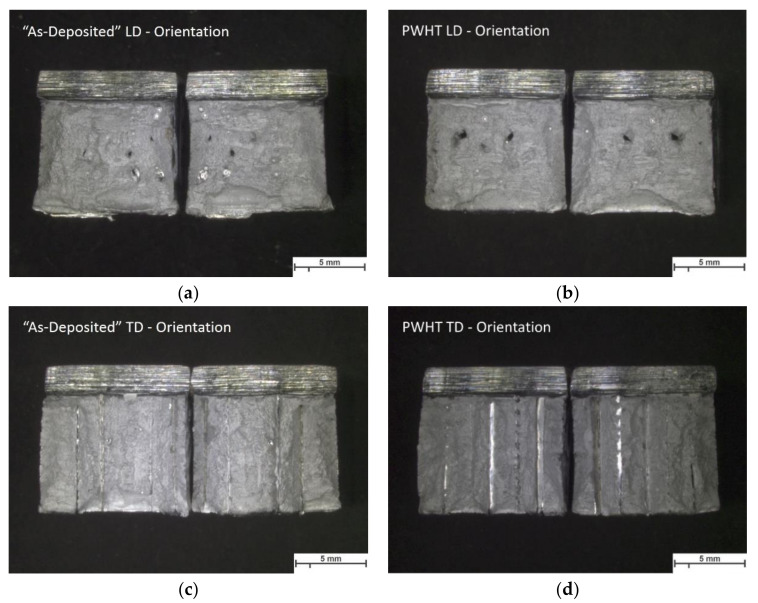
Stereo macrographs of the fractured surfaces in the (**a**,**c**) as-deposited and (**b**,**d**) PWHT condition of the tested longitudinal and transverse orientated Charpy V-notch specimens.

**Figure 11:**
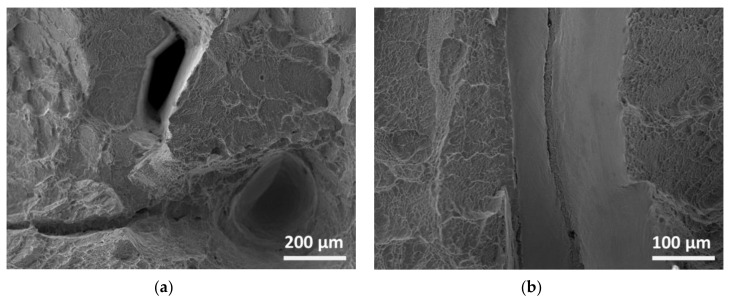
SEM micrographs of the fractured surfaces of Charpy V-notch specimens: (**a**) as-deposited in LD-orientation and (**b**) PWHT in TD-orientation specimens.

The absorbed energy and measured lateral expansion of the tested specimens are listed in Table 6. The results show generally a high impact toughness in the as-deposited condition in comparison to experiments carried out by SLM or EBM [64,65,66] and a slight reduction of the absorbed energy after PWHT. The orientation of the sample affected the impact energy. Specimens with longitudinal (LD) orientation shows higher value than the transverse oriented (TD) samples. Furthermore, a non-isotropic behavior is observed and it might be related to the imperfections produced during the process, as exemplified in Figure 10 and Figure 11.

Stereo micrographs in Figure 10 show irregularities in the fractured surfaces. The fractured surfaces of the LD samples are characterized by randomly distributed and scattered imperfections, mainly related to macro (about 200 μm size) and micro (approx. 5 μm) porous and sharp-edged cavities (Figure 11a). In the case of TD samples, uniform and recurring imperfections in the direction of welding for the entire weld length might be related to a lack of fusion during the process, as observed in Figure 11b.

#### 3.6.3. Hardness Testing

**Figure 12:**
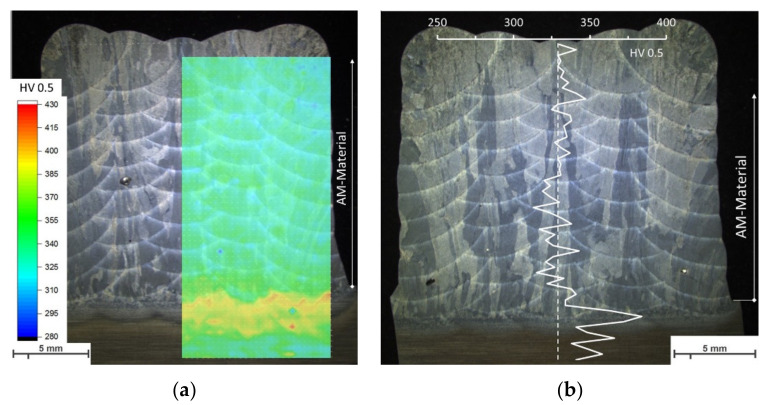
(**a**) Hardness-mapping of transverse cross-section in as-deposited condition and (**b**) hardness line scan in PWHT condition (standard deviation = ±9 HV).

The hardness mapping of the as-deposited condition (Figure 12a) shows an almost uniform distribution of the hardness in the whole transverse cross-section, with an average value of 334 ± 9 HV. The hardness decreases slightly at the fusion lines and the heat-affected zone (HAZ) between the AM block and substrate shows the highest values of hardness (~400 HV). Similar distribution of hardness is observed in PWHT condition, represented in Figure 12b by a line scan from top of the building block to the substrate. The average value for the heat-treated condition is 329 ± 9 HV.

## 4. Discussion

### 4.1. Electron Beam Processing

#### 4.1.1. Dimension of Single Beads

The single track experiments showed that the width and height of the welding beads can be adjusted by the machine parameters. The evaluated parameters for the low input DOE have nearly the same tendencies as Wallace et al. described [9]. Wide beads can be achieved especially at high current, while high beads can be produced by a big amount of wire per unit length.

Sliva et al. [67], Gudenko et al. [68], and Dragunov et al. [69] pointed out the importance of beam oscillation parameters on the weld bead formation. By using concentric beam oscillations, the change of oscillation parameters (e.g., amplitude) together with beam current, the electron beam energy distribution and thus the distribution of energy input in the active zone can be adjusted. In the present study, for all experiments, the beam oscillation parameters (Table 2)—i.e., electron beam distribution—were kept constant on a circular area of a diameter of 4 mm and only the increase of the beam current results in an increase of the energy input by a similar beam distribution. Partial melting of the substrate by the electron beam is required to form the desirable liquid metal bridge material transfer mode between substrate and wire [70,71]. Tang et al. [13] investigated the heat transfer in EBF^3^ and simulated fluid flow of the weld pool by a 3D transient model. In EBF^3^ the high temperature region is restricted to the area of a direct electron beam exposure, showing a steep temperature gradient to its surrounding. An enlargement of the fusion width predicted by simulation of Tang et al. [13] shows a directly proportional relationship to the increase of the beam current, in good agreement with the present study. Taking literature and present study into account, it can be concluded that the melt pool width and therefore maximum weld bead width is predominantly described by beam oscillation parameters (e.g., area of beam exposure i.e., amplitude of beam deflection) as well as the selected beam current. In contrast to the width, the weld bead height is mainly determined by the wire feed rate and the welding speed. For gas metal arc welding (GMAW) process, Xiong et al. [72] observed that the ratio of wire feed rate to welding speed mainly influences the shape of the weld bead profile. Independent of the applied wire-based AM process, the ratio describes the material input per unit length. In EBF^3^ no distinct evaporation of titanium (predominantly evaporation of aluminum [4,52]) and no larger material loss is expected, therefore the composition of the melt pool consists out of the molten substrate/previous layer and fed filler wire. The volume of weld bead reinforcement is therefore determined by the amount of material input per unit length and the ratio of wire feed rate and welding speed. As the weld bead width is not significantly influenced by the velocities, any change of wire feed rate or welding speed (i.e., material input per length) results in a direct change of the weld bead height.

#### 4.1.2. Dilution of Single Bead

In AM, it is desirable to reduce the dilution to improve the efficiency of the stacking process and to avoid a large number of layers and extensive heat input. Within the examined process limits (Table 4), in principle, the lowest dilutions were realized by minimum beam current. A reduction of the beam current not only narrows directly proportional the fusion width, but also the fusion depth and thus the volume of the molten substrate/previous layer [13]. By minimizing the beam current to a threshold value and simultaneously increasing the material input per unit length—i.e., increasing the ratio of wire feed speed to welding speed—a maximum weld bead reinforcement and minimum dilution can be achieved. Though, a minimum melting of the substrate/previous layer is still mandatory for desirable material transfer mode [70,71]. The dilution of 28% in this work was comparatively low since the aim was to achieve a fast build-up process with minimum energy input. However, a minimal dilution and a certain amount of fused metal are required for integrity and for a compensation of wavelike surfaces with valleys between the individual beads of the previous layers. The selected process parameters and overlapping distances enabled a rapid stacking process at a low dilution, whereby bonding defects could not be completely avoided (e.g., Figure 11). It can be assumed that an adjustment of the parameters and avoidance of lack of fusion can further improve mechanical properties.

#### 4.1.3. Overlapping Distance for Multi Track

Since more volumetric AM structures require several tracks per layer and independent of the feed stock, the overlap distance between the tracks is of particular interest for PBF (e.g., [73,74]), but also in wire-based processes (e.g., [10,14]). For arc welding processes there are several models in the literature for describing an optimized overlap in general [43,72,75,76,77,78,79]. The models are based on different theoretical assumptions, which mainly relate to the geometry of the weld bead and related cross-sectional profile. Even when recent models also take the spreading of the weld bead into previously deposited bead and thus changing the geometry, the transferability on weld deposits produced by EBF^3^ is questionable. In EBF^3^, unlike GMAW processes, the material- and energy input is decoupled and material transfer modes, but also heat source models, differ.

To the best of the authors’ knowledge, no general models or recommendations for the overlap distance are proposed in the literature for wire-based additive manufacturing using electron beam so far. For this reason, the present study opted for an experimental approach, where the axial offset of the following track from the adjacent track was varied between 0.55 and 0.75 of the single track width. It produces a surface flatness in conjunction with an acceptable valley formation.

#### 4.1.4. Building AM Block

**Figure 13:**
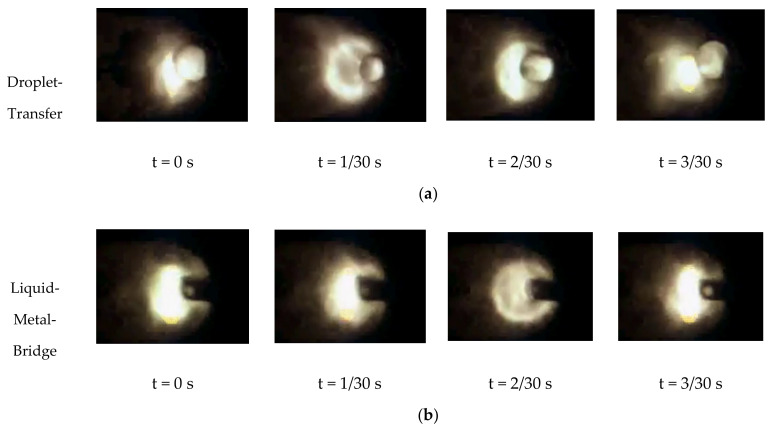
Material transfer modes in electron beam additive manufacturing recorded via CCT camera: (**a**) droplet transfer and (**b**) liquid metal bridge transfer.

Since the process takes place in a vacuum atmosphere, the heat is slower dissipated by heat conduction and thermal radiation, without the contribution of convection like in WAAM. The transient heat flux is distorted by the increasing number of layers, and the ratio of heat dissipated by thermal radiation to heat conduction increases. Since the temperature increases, weld bead geometry changes and tends to widen and flatten [80]. The changing weld bead height and deviation from the nominal values accumulate layer by layer. In wire-based AM processes (e.g., EBAM) where energy and material input are separated, the exact alignment of the filler material to the previous layer/substrate determines the material transfer mode as predominant [70,71]. If the relative position of the wire tip to the previous layer/substrate increases when increasing the number of layers, the material transfer mode changes from a preferable liquid metal bridge transfer (Figure 13b) to a droplet transfer (Figure 13a), promoting spatter formation and irregular metal deposition [10].

By continuously reducing the beam current (i.e., power input) every second layer, the increasing preheating effect can be overcome, and a constant growth rate of 1.64 mm per new layer can be ensured. A uniform growth rate per layer is decisive for setting up an automated setup and guarantee stable material transfer.

The energy input and welding sequence per layer for Ti-6Al-4V affect the microstructure and related properties [81] due to the process’ cooling rates. For gas metal arc welding, Stockinger et al. [50] and Plangger et al. [51] showed that an alternating welding sequence results in a uniform AM structure with homogenous properties for martensitic steels in transverse cross-sections. A similar approach was chosen and by alternating weld bead sequence, the energy input and heat can be distributed more uniformly. The morphology of the microstructure was almost homogenous in the whole transverse cross-section and also no changes in hardness for the AM material could be observed (Figure 12).

### 4.2. Effect of the Microstructure on the Mechanical Properties

**Figure 14:**
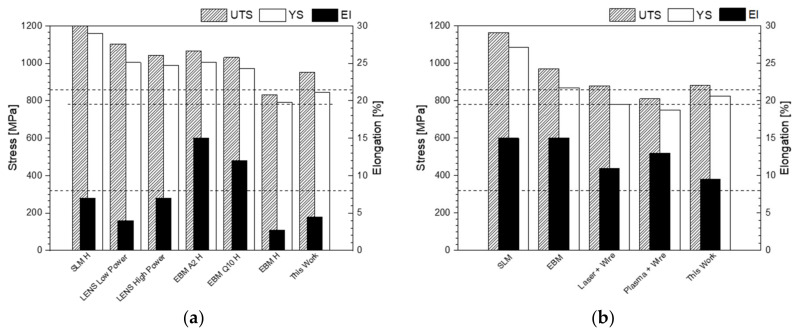
Comparison of the results obtained in the present work with other process. (**a**) As deposited condition is compared with values from LENS, ABM A2 H, and EBM Q 10 processes referred by Zhai et al. [29], SLM reviewed by Liu et al. [81] and EBM H by Edwards et al. [82]. (**b**) after PWHT is compared with a summary of processes given by Greitmeier [15] and Liu et al. [81].

The tensile testing showed that the achieved results are in the low range of typical values for this alloy. A comparison to additive manufactured parts with other processes from literature is shown in Figure 14. This difference in the values is related mainly to the microstructure built during the process. In general, as deposited conditions of SLM components have shown the highest values of tensile strength due to the fully retained martensite [20,81]. Buildings by LENS with different energies show mainly α´martensite structure mixed with some acicular α phase, while EBM powder-based process retained very fine α laths due to longer time exposure at 650–750 °C during the manufacturing [29]. The lower values observed by Edwards et al. [82] are related to a slow cooling during the process and partial stabilization of the α phase. The apparent martensitic form observed in this work (Figure 7) has been decomposed into α + β during the deposition. The building sequence induces by the heat input a short time annealing effect on the microstructure, reducing the hardness to values closer to the PWHT (Figure 14). Although moderate/high cooling rate is expected in this process (~150–400 °C/s, as reported in the Figure S2), the high energy input of this process and the bad thermal conductivity of Ti-6Al-4V leads to keep warm temperatures in the last deposited layer [30]. On the other hand, the presence of residual stresses in the as deposited condition, might be related not only to the volume distortion provoked during the cooling but also to the partial transformation of the apparent martensitic structure, in agreement with observations in laser metal deposited process [62]. The deposited material shows lower strength values than typical martensitic structure in wrought material, but with the same elongation [23,83].

Although there is no significant changes in the tensile strength in comparison with wire-feed processes [15] after stress relief treatment (PWHT), the values of strength are substantial lower than the observed in SLM process [81]. This difference might be related to the α lath size reached during the annealing treatment [81]. Therefore, further investigation to optimize the microstructure by thermal treatments is necessary to obtain the desirable mechanical properties.

## 5. Conclusions

In this work, the use of wire-based additive by electron beam process for Ti-6Al-4V was investigated. Suitable parameters for building single and robust walls were found and the microstructure in as deposited and after heat treatment was correlated with the mechanical properties. The findings can be summarized as follows:

- During the building of the wall, the bead shape is mainly affected by the beam current, the weld-velocity, and the feeding rate of the wire.
- A fast build-up process with minimum energy input guaranteed a dilution of 28%, which facilitate an optimal overlapping distance to reduce the wave-like surface.
- The use of a symmetric welding sequence with an overlapping distance of 70–75% of the bead-width permits a flat surface and a linear growth rate of the block.
- The chemical composition of the AM material shows only evaporation losses of aluminum in the range of approx. 1 wt %.
- As-deposited condition showed a mixture of finer α and martensitic structure within the coarser columnar prior β grains, providing a low tensile strength compared to similar additive manufacturing processes.

The lack of fusion observed during impact tests must be improved in further investigations for a precise relationship of microstructure and mechanical properties.

## Figures and Tables

**Table 1:** Measured chemical composition of applied titanium alloy wire AWS A5.16 ER Ti5 (EN ISO 24034); n.m.: not measured.

Material	Al (wt. %)	V (wt. %)	Fe (wt. %)	Ti (wt. %)	C (wt. %)	N (wt. %)	O (wt. %)
Solid Wire	6.36	3.48	0.11	bal.	0.018	<0.005	n.m.

**Table 2:** Summary of input and beam oscillation (bop) parameters for welding and adjustment of wire feed unit.

Attribute	Abbreviation	Unit	Values
Acceleration voltage	*U_acc_*	kV	90
Beam current	*I_beam_*	mA	17.5–46.7
Welding speed	*v_weld_*	mm/s	9.0–11.0
Wire feed rate	*V_wire_*	m/min	2.7–3.9
Feed angle	*α_feed_*	-	55
Focal point	*f_p_*	-	Substrate Surface
Beam figure (bop)	*-*	-	Concentric Circles
Frequency (bop)	*f*	Hz	1000
Amplitude of deflection (bop)	*x*,*y*	mm	Ø 4

**Table 3:** Factor levels and combinations of full factorial design (FFD); moderate and higher values of deposition rates; and power input.

Factor		Unit	Heat Input			
			Moderate		High	
			Low	High	Low	High
Beam current	*I_beam_*	mA	17.5	21.4	31.1	46.7
Welding speed	*v_weld_*	mm/s	9.0	11.0	9.0	11.0
Wire feed rate	*V_wire_*	m/min	2.7	3.3	3.3	3.9

**Table 4:** Database and settings to Figure 3.

Figure 3	Beam Current	Welding Speed	Wire Feed Rate	Material Input per Length	Dilution
	(mA)	(mm/s)	(m/min)	(-)	(%)
(**a**)	17.5	11	2.7	4.1	45
(**b**)	21.4	11	3.3	5.0	45
(**c**)	21.4	9	2.7	5.0	52
(**d**)	17.5	9	3.3	6.1	28

**Table 5:** Chemical composition of AM bulk material.

Material	Al (wt. %)	V (wt. %)	Fe (wt. %)	Ti (wt. %)	C (wt. %)	N (wt. %)	O (wt. %)
AM block no. 1	5.47	3.39	0.11	bal.	0.017	<0.005	0.11
AM block no. 2	5.49	3.46	0.10	bal.	0.019	<0.005	0.11

**Table 6:** Results of Charpy V-notch impact testing in longitudinal (LD) and transverse direction (TD) for as-deposited and PWHT condition.

Sample Orientation	As-Deposited		PWHT	
	Impact Energy (J)	Lateral Expansion (mm)	Impact Energy (J)	Lateral Expansion (mm)
LD	54	0.6	45	0.4
TD	40	0.3	34	0.3

## Supplementary Materials

The following is available online at https://www.mdpi.com/1996-1944/13/15/3310/s1, Figure S1: (a) temperature distribution for a single track deposition in electron beam additive manufacturing and (b) derived cooling rates, Figure S2: (a) exponential fit of the measured values and (b) derived cooling rates for a single track deposition via electron beam additive manufacturing.

## References

[1] ISO 17296-2:2015 (2015). Additive Manufacturing—General Principles—Part 2: Overview of Process Categories and Feedstock.

[2] Taminger K., Hafley R. Electron beam freeform fabrication: A rapid metal deposition process. Proceedings of the 3rd Annual Automotive Composites Conference.

[3] Wanjara P., Watanabe K., De Formanoir C., Yang Q., Bescond C., Godet S., Brochu M., Nezaki K., Gholipour J., Patnaik P. (2019). Titanium alloy repair with wire-feed electron beam additive manufacturing technology. Adv. Mater. Sci. Eng..

[4] Xu J., Zhu J., Fan J., Zhou Q., Peng Y., Guo S. (2019). Microstructure and mechanical properties of Ti-6Al-4V alloy fabricated using electron beam freeform fabrication. Vacuum.

[5] Savchenko N.L. (2019). Structure and phase composition of Ti-6Al-4V alloy made by additive manufacturing technology. AIP Conf. Proc..

[6] Taminger K., Hafley R. Electron beam freeform fabrication for cost effective near-net shape manufacturing. Proceedings of the NATO AVT-139 Specialists’ Meeting—Cost Effective Manufacture via Net Shape Processing.

[7] Schultz H. (1994). Electron Beam Welding.

[8] Sciaky Inc. Electron Beam Additive Manufacturing. http://www.sciaky.com/additive-manufacturing/electron-beam-additive-manufacturing-technology.

[9] Wallace T., Bey K., Taminger K., Hafley R. A design of experiments approach defining the relationships between processing and microstructure for Ti-6Al-4V. Proceedings of the 15th Solid Freeform Fabrication Symposium.

[10] Fuchs J., Schneider C., Enzinger N. (2018). Wire-based additive manufacturing using an electron beam as heat source. Weld. World.

[11] Gockel J., Beuth J., Taminger K. (2014). Integrated control of solidification microstructure and melt pool dimensions in electron beam wire feed additive manufacturing of Ti-6Al-4V. Addit. Manuf..

[12] Gockel J., Fox J., Beuth J., Hafley R. (2015). Integrated melt pool and microstructure control for Ti-6Al-4V thin wall additive manufacturing. Mater. Sci. Technol..

[13] Tang Q., Pang S., Chen B., Suo H., Zhou J. (2014). A three dimensional transient model for heat transfer and fluid flow of weld pool during electron beam freeform fabrication of Ti-6-Al-4-V alloy. Int. J. Heat Mass Transf..

[14] Ding D., Pan Z., Cuiuri D., Li H., van Duin S., Larkin N. (2016). Bead modelling and implementation of adaptive MAT path in wire and arc additive manufacturing. Robot. Comput. Integr. Manuf..

[15] Greitemeier D. (2016). Untersuchung der Einflussparameter Auf die Mechanischen Eigenschaften von Additiv Gefertigtem TiAl6V4.

[16] Fadida R., Rittel D., Shirizly A. (2015). Dynamic mechanical behavior of additively manufactured Ti_6_Al_4_V with controlled voids. J. Appl. Mech. Trans..

[17] Kasperovich G., Haubrich J., Gussone J., Requena G. (2016). Correlation between porosity and processing parameters in TiAl_6_V_4_ produced by selective laser melting. Mater. Des..

[18] Wu B., Pan Z., Ding D., Cuiuri D., Li H., Xu J., Norrish J. (2018). A review of the wire arc additive manufacturing of metals: Properties, defects and quality improvement. J. Manuf. Process..

[19] DebRoy T., Wei H.L., Zuback J.S., Mukherjee T., Elmer J.W., Mileweski J.O., Beese A.M., Wilson-Heid A., De A., Zhang W. (2018). Additive manufacturing of metallic components—Process, structure and properties. Prog. Mater. Sci..

[20] Agius D., Kourousis K., Wallbrink C. (2018). A review of the as-built SLM Ti-6Al-4V mechanical properties towards achieving fatigue resistant designs. Metals.

[21] Tiferet E., Ganor M., Zolotaryov D., Garkun A., Hadjadj A., Chonin M., Ganor Y., Noiman D., Halevy I., Tevet O. (2019). Mapping the tray of electron beam melting of Ti-6Al-4V: Properties and microstructure. Materials.

[22] Leyens C., Peters M. (2003). Titanium and Titanium Alloys: Fundamentals and Applications.

[23] Lütjering G., Williams J. (2007). Titanium.

[24] Zhang L.C., Liu Y., Li S., Hao Y. (2017). Additive manufacturing of titanium alloys by electron beam melting: A review. Adv. Eng. Mater..

[25] Roberts I.A., Wang C.J., Esterlein R., Stanford M., Mynors D.J. (2009). A three-dimensional finite element analysis of the temperature field during laser melting of metal powders in additive layer manufacturing. Int. J. Mach. Tools Manuf..

[26] Li Y., Gu D. (2014). Parametric analysis of thermal behavior during selective laser melting additive manufacturing of aluminum alloy powder. Mater. Des..

[27] Murr L.E., Esquivel E.V., Quinones S.A., Gaytan S.M., Lopez M.I., Martinez E.Y., Medina F., Hernandez D.H., Martinez E., Stafford S.W. (2008). Microstructure and mechanical properties of electron beam-rapid manufactured Ti-6Al-4V biomedical prototypes compared to wrought Ti-6Al-4V. Mater. Charact..

[28] Tan X., Kok Y., Tan Y.J., Descoins M., Mangelinck D., Tor S.B., Leong K.F., Chua C.K. (2015). Graded microstructure and mechanical properties of additive manufactured Ti-6Al-4V via electron beam melting. Acta Mater..

[29] Zhai Y., Galarraga H., Lados D.A. (2016). Microstructure, static properties, and fatigue crack growth mechanisms in Ti-6Al-4V fabricated by additive manufacturing: LENS and EBM. Eng. Fail. Anal..

[30] Fachinotti V.D., Cardona A., Baufeld B., Van der Biest O. (2012). Finite-element modelling of heat transfer in shaped metal deposition and experimental validation. Acta Mater..

[31] Al-Bermani S.S., Blackmore M.L., Zhang W., Todd I. (2010). The origin of microstructural diversity, texture, and mechanical properties in electron beam melted Ti-6Al-4V. Metall. Mater. Trans..

[32] Brandl E., Schoberth A., Leyens C. (2012). Morphology, microstructure, and hardness of titanium (Ti-6Al-4V) blocks deposited by wire-feed additive layer manufacturing (ALM). Mater. Sci. Eng. A.

[33] Baufeld B., Brandl E., Van der Biest O. (2011). Wire based additive layer manufacturing: Comparison of microstructure and mechanical properties of Ti-6Al-4V components fabricated by laser-beam deposition and shaped metal deposition. J. Mater. Process. Technol..

[34] Wang J., Lin X., Li J., Hu Y., Zhou Y., Wang C., Li Q., Huang W. (2019). Effects of deposition strategies on macro/microstructure and mechanical properties of wire and arc additive manufactured Ti–6Al–4V. Mater. Sci. Eng. A.

[35] Wang J., Lin X., Wang J., Yang H., Zhou Y., Wang C., Li Q., Huang W. (2018). Grain morphology evolution and texture characterization of wire and arc additive manufactured Ti-6Al-4V. J. Alloys Compd..

[36] DVS 2713:2016-04 (2016). Schweißen von Titanwerkstoffen Werkstoffe—Prozesse—Fertigung—Prüfung und Bewertung von Schweißverbindungen.

[37] DIN 65084:1990-04 (1990). Luft-und Raumfahrt Wärmebehandlung von Titan und Titan-Knetlegierungen.

[38] DIN EN ISO 6892-1:2017-02 (2017). Metallische Werkstoffe—Zugversuch—Teil 1: Prüfverfahren bei Raumtemperatur.

[39] DIN EN ISO 148-1:2017-05 (2017). Metallische Werkstoffe—Kerbschlagbiegeversuch nach Charpy—Teil 1: Prüfverfahren.

[40] DIN EN ISO 6507-1:2016-08 (2016). Metallische Werkstoffe—Härteprüfung nach Vickers—Teil 1: Prüfverfahren.

[41] Larson A.C., von Dreele R.B. (2000). General Structure Analysis System (GSAS).

[42] Fahrenwaldt H.J., Schuler V. (2006). Praxiswissen Schweißtechnik.

[43] Suryakumar S., Karunakaran K.P., Bernard A., Chandrasekhar U., Raghavender N., Sharma D. (2011). Weld bead modeling and process optimization in Hybrid Layered Manufacturing. CAD Comput. Aided Des..

[44] Ding D., Pan Z., Cuiuri D., Li H. (2015). A multi-bead overlapping model for robotic wire and arc additive manufacturing (WAAM). Robot. Comput. Integr. Manuf..

[45] Ding D., Pan Z., Cuiuri D., Li H., Larkin N. (2016). Adaptive path planning for wire-feed additive manufacturing using medial axis transformation. J. Clean. Prod..

[46] Jin Y., He Y., Fu J., Gan W., Lin Z. (2014). Optimization of tool-path generation for material extrusion-based additive manufacturing technology. Addit. Manuf..

[47] Jin Y., He Y., Fu J., Zhang A., Du J. (2017). A non-retraction path planning approach for extrusion-based additive manufacturing. Robot. Comput. Integr. Manuf..

[48] Ding D., Pan Z., Cuiuri D., Li H. (2015). A practical path planning methodology for wire and arc additive manufacturing of thin-walled structures. Robot. Comput. Integr. Manuf..

[49] Ding D., Shen C., Pan Z., Cuiuri D., Li H., Larkin N., van Duin S. (2016). Towards an automated robotic arc-welding-based additive manufacturing system from CAD to finished part. Comput. Des..

[50] Stockinger J., Wiednig C.A., Enzinger N., Sommitsch C., Huber D., Stockinger M. Additive Manufacturing via Cold Metal Transfer. Proceedings of the Metal Additive Manufacturing Conference.

[51] Plangger J., Schabhüttl P., Vuherer T., Enzinger N. (2019). CMT additive manufacturing of a high strength steel alloy for application in crane construction. Metals.

[52] Chen T., Pang S., Tang Q., Suo H., Gong S. (2016). Evaporation Ripped Metallurgical Pore in Electron Beam Freeform Fabrication of Ti-6-Al-4-V. Mater. Manuf. Process..

[53] Semiatin S.L., Ivanchenko V.G., Akhonin S.V., Ivasishin O.M. (2004). Diffusion models for evaporation losses during electron-beam melting of alpha/beta-titanium alloys. Metall. Mater. Trans. B Process. Metall. Mater. Process. Sci..

[54] Juechter V., Scharowsky T., Singer R.F., Körner C. (2014). Processing window and evaporation phenomena for Ti-6Al-4V produced by selective electron beam melting. Acta Mater..

[55] Zhang G., Chen J., Zheng M., Yan Z., Lu X., Lin X., Huang W. (2020). Element vaporization of Ti-6AL-4V alloy during selective laser melting. Metals.

[56] Sequeira Almeida P.M., Williams S. Innovative process model of Ti-6Al-4V additive layer manufacturing using cold metal transfer (CMT). Proceedings of the 21st Annual International Solid Freeform Fabrication Symposium.

[57] Bermingham M.J., StJohn D.H., Krynen J., Tedman-Jones S., Dargusch M.S. (2019). Promoting the columnar to equiaxed transition and grain refinement of titanium alloys during additive manufacturing. Acta Mater..

[58] Åkerfeldt P., Antti M.-L., Pederson R. (2016). Influence of microstructure on mechanical properties of laser metal wire-deposited Ti-6Al-4V. Mater. Sci. Eng. A.

[59] Ho A., Zhao H., Fellowes J.W., Martina F., Davis A.E., Prangnell P.B. (2019). On the origin of microstructural banding in Ti-6Al4V wire-arc based high deposition rate additive manufacturing. Acta Mater..

[60] Haubrich J., Gussone J., Barriobero-Vila P., Kürnsteiner P., Jägle E.A., Raabe D., Schell N., Requena G. (2019). The role of lattice defects, element partitioning and intrinsic heat effects on the microstructure in selective laser melted Ti-6Al-4V. Acta Mater..

[61] Daymond M.R., Bourke M.A.M., von Dreele R.B. (1999). Use of Rietveld refinement to fit a hexagonal crystal structure in the presence of elastic and plastic anisotropy. J. Appl. Phys..

[62] Lundbäck A., Pederson R., Colliander M.H., Brice C., Steuwer A., Heralic A., Buslaps T., Lindgren L.-E. (2016). Modeling and experimental measurement with synchrotron radiation of residual stresses in laser metal deposited Ti-6Al-4V. Proceedings of the 13th World Conference on Titanium, San Diego, CA, USA, 16–20 August 2015.

[63] ASTM F136-13 (2013). Standard Specification for Wrought Titanium-6Aluminum-4Vanadium ELI (Extra Low Interstitial) Alloy for Surgical Implant Applications (UNS R56401).

[64] Grell W.A., Solis-Ramos E., Clark E., Lucon E., Garboczi E.J., Predecki P.K., Loftus Z., Kumosa M. (2017). Effect of powder oxidation on the impact toughness of electron beam melting Ti-6Al-4V. Addit. Manuf..

[65] Yasa E., Deckers J., Kruth J.-P., Rombouts M., Luyten J. (2010). Charpy impact testing of metallic selective laser melting parts. Virtual Phys. Prototyp..

[66] Wu M.W., Lai P.H. (2016). The positive effect of hot isostatic pressing on improving the anisotropies of bending and impact properties in selective laser melted Ti-6Al-4V alloy. Mater. Sci. Eng. A.

[67] Sliva A.P., Dragunov V.K., Terentyev E.V., Goncharov A.L. (2018). EBW of aluminium alloys with application of electron beam oscillation. Proc. J. Phys. Conf. Ser..

[68] Gudenko A.V., Sliva A.P. (2018). Influence of electron beam oscillation parameters on the formation of details by electron beam metal wire deposition method. Proc. J. Phys. Conf. Ser..

[69] Dragunov V.K., Goryachkina M.V., Gudenko A.V., Sliva A.P., Shcherbakov A.V. Investigation of the optimal modes of electron-beam wire deposition. Proceedings of the IOP Conference Series: Materials Science and Engineering.

[70] Zhao J., Zhang B., Li X., Li R. (2015). Effects of metal-vapor jet force on the physical behavior of melting wire transfer in electron beam additive manufacturing. J. Mater. Process. Technol..

[71] Hu R., Luo M., Liu T., Liang L., Huang A., Trushnikov D., Karunakaran K.P., Pang S. (2018). Thermal fluid dynamics of liquid bridge transfer in laser wire deposition 3D printing. Sci. Technol. Weld. Join..

[72] Xiong J., Zhang G., Gao H., Wu L. (2013). Modeling of bead section profile and overlapping beads with experimental validation for robotic GMAW-based rapid manufacturing. Robot. Comput. Integr. Manuf..

[73] Dong Z., Liu Y., Wen W., Ge J., Liang J. (2019). Effect of hatch spacing on melt pool and as-built quality during selective laser melting of stainless steel: Modeling and experimental approaches. Materials.

[74] Wang P., Nai M.L.S., Sin W.J., Lu S., Zhang B., Bai J., Song J., Wei J. (2019). Effect of overlap distance on the microstructure and mechanical properties of in situ welded parts built by electron beam melting process. J. Alloys Compd..

[75] Cao Y., Zhu S., Liang X., Wang W. (2011). Overlapping model of beads and curve fitting of bead section for rapid manufacturing by robotic MAG welding process. Robot. Comput. Integr. Manuf..

[76] Li Y., Han Q., Zhang G., Horváth I. (2018). A layers-overlapping strategy for robotic wire and arc additive manufacturing of multi-layer multi-bead components with homogeneous layers. Int. J. Adv. Manuf. Technol..

[77] Nguyen L., Buhl J., Bambach M. (2020). Multi-bead overlapping models for tool path generation in wire-arc additive manufacturing processes. Procedia Manuf..

[78] Li Y., Sun Y., Han Q., Zhang G., Horváth I. (2018). Enhanced beads overlapping model for wire and arc additive manufacturing of multi-layer multi-bead metallic parts. J. Mater. Process. Technol..

[79] Aiyiti W., Zhao W., Lu B., Tang Y. (2006). Investigation of the overlapping parameters of MPAW-based rapid prototyping. Rapid Prototyp. J..

[80] Nikam S.H., Jain N.K. (2018). Finite element simulation of pre-heating effect on melt pool size during micro-plasma transferred arc deposition process. IOP Conf. Ser. Mater. Sci. Eng..

[81] Liu S., Shin Y.C. (2019). Additive manufacturing of Ti6Al4V alloy: A review. Mater. Des..

[82] Edwards P., O’Conner A., Ramulu M. (2013). Electron beam additive manufacturing of titanium components: Properties and performance. J. Manuf. Sci. Eng..

[83] Chong Y., Bhattacharjee T., Yi J., Shibata A., Tsuji N. (2017). Mechanical properties of fully martensite microstructure in Ti-6Al-4V alloy transformed from refined beta grains obtained by rapid heat treatment (RHT). Scr. Mater..

