# Supplementary material for: Wire-Based Additive Manufacturing of Ti-6Al-4V Using Electron Beam Technique
Source: Materials (Basel). 2020 Jul 24;13(15):3310. doi: 10.3390/ma13153310 (PMC7435555; doi:10.3390/ma13153310)
Supplement: Supplementary file 1 [file materials-13-03310-s001.pdf]

materials

Supplementary Materials

Wire-Based Additive Manufacturing of Ti-6Al-4V Using Electron Beam Technique

Contactless temperature measurement in electron beam additive manufacturing[1]

The temperature profile was recorded with a near infrared (NIR) camera Optris PI 1M (Optris GmbH, Berlin, Germany), which covers the spectral range from 0.85–1.1 µm and the temperature range from 575–1800 °C. An emissivity ε of 0.30 was determined based on the weld bead width ($T_{solidus}$ = $T_{W/2}$) and amplitude of beam deflection ($T_{Maximum}$ = $T_{Amplitude\ of\ Deflection}$). The evaluated emissivity correlates with the values proposed in the literature, which comprise between 0.3–0.5 for high temperature [1,2]. The NIR camera features 764 × 480 pixels with a frame rate of 8 Hz. Images were recorded for single track deposition in the first layer and the region of interest (ROI) was exported with a size of 140 × 60 pixels. The data were visualized using the software MATLAB R2017a (Version 9.2, MathWorks, Natick, MA, USA), illustrating the temperature profile described by isotherms and the cooling rates as derived from the temperature profile (Figure S1).

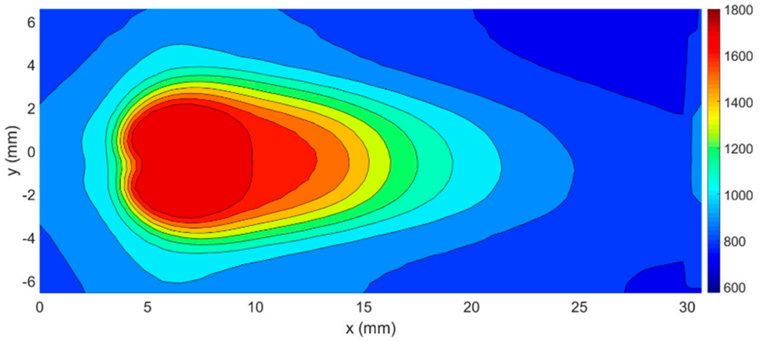

(**a**)

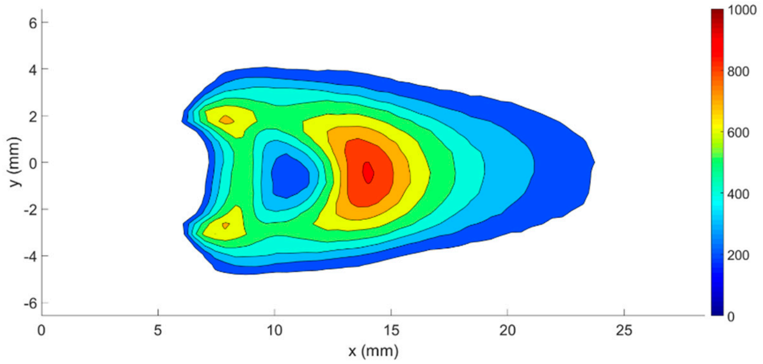

(**b**)

Figure S1. (**a**) Temperature distribution for a single track deposition in electron beam additive manufacturing and (**b**) derived cooling rates.

Based on the measurements, temperature profile can be described by exponential fit and its deviation as a function of time describes the cooling rate (Figure S2). In the temperature range of 850–

[1] Information was taken from a manuscript in progress (July 2020)

1000°C (α + β field) cooling rates of approx. 150–400 °C/s can be expected. According to schematic continuous cooling transformation diagram for Ti-6Al-4V [3], transformation β → α′ + α can take place in the present study.

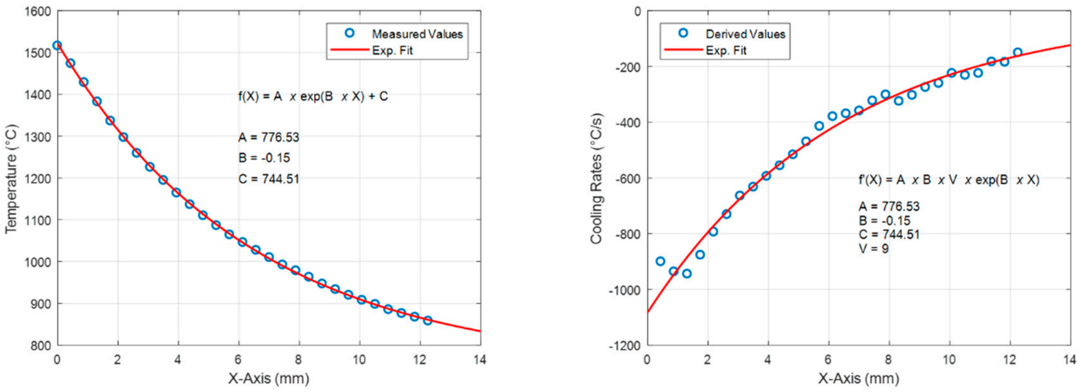

(a) (b)

Figure S1. (**a**) Exponential fit of the measured values and (**b**) derived cooling rates for a single track deposition via electron beam additive manufacturing

Reference

1. Michels, W.C.; Wilford, S. The physical properties of titanium. I. emissivity and resistivity of the commercial metal. *J. Appl. Phys.* **1949**, *20*, 1223–1226. doi:10.1063/1.1698312
2. Bradshaw, F.J. The optical emissivity of titanium and zirconium. *Proc. Phys. Soc. Sect. B* **1950**, *63*, 573–577. doi:10.1088/0370-1301/63/8/304
3. Ahmed, T.; Rack, H.J. Phase transformations during cooling in α + β titanium alloys. *Mater. Sci. Eng. A* **1998**, *243*, 206–211. doi:10.1016/S0921-5093(97)00802-2

© 2020 by the author. Licensee MDPI, Basel, Switzerland. This article is an open access article distributed under the terms and conditions of the Creative Commons Attribution (CC BY) license (http://creativecommons.org/licenses/by/4.0/).
